# B-Cell Maturation Antigen (BCMA) as a Target for New Drug Development in Relapsed and/or Refractory Multiple Myeloma

**DOI:** 10.3390/ijms21155192

**Published:** 2020-07-22

**Authors:** Hanley N. Abramson

**Affiliations:** Department of Pharmaceutical Sciences, Wayne State University, Detroit, MI 48202, USA; ac2531@wayne.edu

**Keywords:** myeloma, BCMA, bispecific T-cell engager, antibody-drug conjugates, chimeric antigen receptor T-cells, belantamab mafodotin, idecabtagene vicleucel, JNJ-68284528

## Abstract

During the past two decades there has been a major shift in the choice of agents to treat multiple myeloma, whether newly diagnosed or in the relapsed/refractory stage. The introduction of new drug classes, such as proteasome inhibitors, immunomodulators, and anti-CD38 and anti-SLAMF7 monoclonal antibodies, coupled with autologous stem cell transplantation, has approximately doubled the disease’s five-year survival rate. However, this positive news is tempered by the realization that these measures are not curative and patients eventually relapse and/or become resistant to the drug’s effects. Thus, there is a need to discover newer myeloma-driving molecular markers and develop innovative drugs designed to precisely regulate the actions of such putative targets. B cell maturation antigen (BCMA), which is found almost exclusively on the surfaces of malignant plasma cells to the exclusion of other cell types, including their normal counterparts, has emerged as a specific target of interest in this regard. Immunotherapeutic agents have been at the forefront of research designed to block BCMA activity. These agents encompass monoclonal antibodies, such as the drug conjugate belantamab mafodotin; bispecific T-cell engager strategies exemplified by AMG 420; and chimeric antigen receptor (CAR) T-cell therapeutics that include idecabtagene vicleucel (bb2121) and JNJ-68284528.

## 1. Introduction

Multiple myeloma (MM) is a hematological cancer characterized by clonal plasma cell proliferation in the bone marrow along with high levels of monoclonal immunoglobulins in the blood and/or urine. Ranking behind non-Hodgkin’s lymphoma, MM is the second most common blood cancer and the 14th most prevalent cancer overall. It is estimated that in 2020 a total of 32,270 (54.3% male) new cases of the disease will be diagnosed and be responsible for 12,830 deaths in the U.S. [1]. Active MM, which is accompanied by a tetrad of symptoms, generally abbreviated CRAB—hypercalcemia, renal insufficiency, anemia, and bone lesions—often is preceded by an asymptomatic phase known as monoclonal gammopathy of undetermined significance (MGUS). Progression from MGUS to MM, which carries a risk of about 1% per year [2], may also include another asymptomatic state known as smoldering myeloma [3]. The most recent pertinent guidelines for the diagnosis and treatment of MM have been issued by the National Comprehensive Cancer Network (NCCN) [4].

The therapy of MM has seen remarkable progress over the past half century. Beginning in the mid-1960s and continuing for more than three decades, alkylating agents, principally melphalan and cyclophosphamide, often accompanied by corticosteroids, were considered standard therapy for the disease. Starting in the 1990s, treatment protocols for the disease were augmented by autologous stem cell transplantation (ASCT). This established paradigm shifted dramatically starting in the late 1990s with the discovery of thalidomide′s immunomodulatory actions that conferred remarkable anti-myeloma properties on this formerly ignominious agent. This was followed by the mechanistically related lenalidomide in 2005 and later (2013) pomalidomide. Furthermore, the discovery of the anti-myeloma activity of the proteasome inhibitor bortezomib in 2003, subsequently followed by carfilzomib and ixazomib, provided substantive additions to the armamentarium available to fight the disease. In 2015, in another remarkable turn of events, the Food and Drug Administration (FDA) approved two monoclonal antibodies (mAbs)—daratumumab and elotuzumab—for treating MM. Both target glycoproteins found on the surface of MM cells, CD38 and SLAMF7, respectively. Another anti-CD38 mAb, isatuximab-irfc, was approved by the FDA in 2020. Rounding out the currently FDA-approved treatment modalities for MM are the pan-histone deacetylase inhibitor panobinostat (2015) and the nuclear export inhibitor selinexor (2019). The success of these therapeutic advances over the past four decades is attested to by the more than doubling of the disease’s five-year survival rate, from 24.5% in 1975–77 to 55.1% in 2010–2016 [5]. Nevertheless, MM remains largely incurable and relapse and refractoriness to treatment continue as major problems [4], spurring the search for newer molecular targets and discovery of drugs exquisitely designed to modulate the actions of these targets.

## 2. The BAFF/APRIL/BCMA Axis

**Figure 1:**
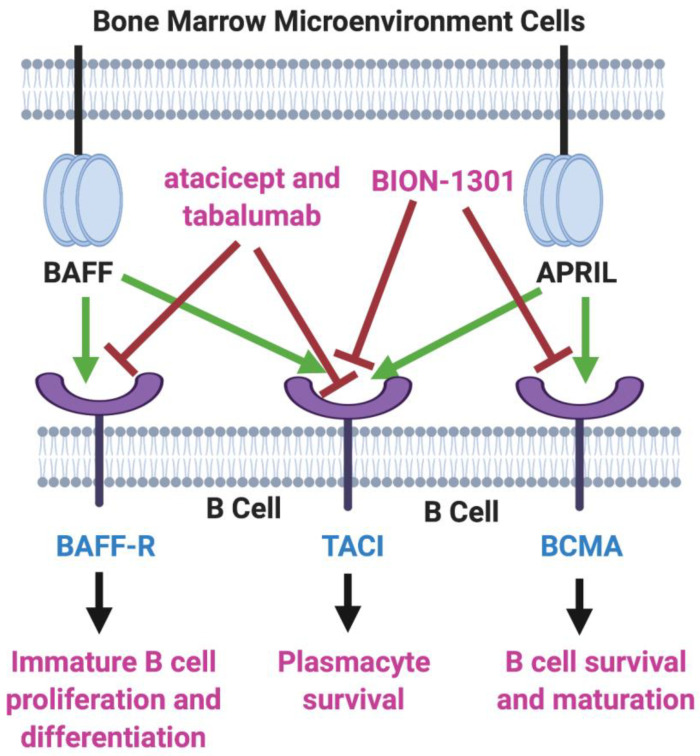
BAFF/APRIL/BCMA Axis. Tumor necrosis factor (TNF) family members BAFF (B-cell activating factor) and APRIL (a proliferation-inducing ligand) are cytokines secreted in the bone marrow microenvironment that play key roles in B cell development, as well as in supporting the viability and proliferation of plasma cells in multiple myeloma. Both BAFF and APRIL serve as ligands for two receptors on the myeloma cell surface—transmembrane activator and calcium modulator and cyclophilin ligand interactor (TACI) and B-cell maturation antigen (BCMA). BAFF also binds to BAFF-R, another myeloma cell receptor. The receptor blockers shown, atacicept, tabalumab, and BION-1301, have been studied in MM patients but have failed to provide evidence of efficacy. On the other hand, inhibitors of BCMA have demonstrated great potential in the therapy of MM. Created with BioRender.com.

B-cell activating factor (BAFF; BLyS; TALL-1) and APRIL (a proliferation-inducing ligand) are two homologous members of the tumor necrosis factor (TNF) superfamily [6,7] that have received much recent attention for their roles in the pathology of lupus erythematosus, rheumatoid arthritis, and other autoimmune diseases [8,9]. There also is evidence that the production of both of these cytokines in the bone marrow microenvironment plays a key role in the viability and proliferation of myeloma cells [10]. Moreover, MM disease progression and prognosis have been linked with BAFF and APRIL serum levels [11]. Both BAFF and APRIL serve as ligands for two TNF receptor family members located on the myeloma cell surface—transmembrane activator and calcium modulator and cyclophilin ligand interactor (TACI) and B-cell maturation antigen (BCMA). In addition, BAFF binds to a third myeloma cell receptor, BAFF-R (Figure 1).

Two inhibitors of both BAFF and APRIL, atacicept and tabalumab, each have been studied in several immune-related conditions, including MM, but have failed to exhibit substantial efficacy in any trials [12,13]. Moreover, BION-1301, a humanized anti-APRIL antibody, that had been considered a possibility for clinical development in MM has been dropped from further consideration in myeloma due to failure to achieve objective responses in a phase I study (NCT03340883) [14].

However, the bulk of attention on the BAFF/APRIL/BCMA axis in MM has been focused on BCMA as a major target of interest, particularly in three immunotherapy fronts: as a mAb (both naked and drug-conjugated); as a component of the bispecific T-cell engager (BiTE) strategy; and in conjunction with chimeric antigen receptor (CAR) T-cell therapy.

## 3. BCMA

BCMA (CD269; TNFRSF17), also a member of the TNF receptor superfamily, was first identified in the early 1990s [15,16,17,18]. Expression of this 184-amino acid glycoprotein plays a major role in B-cell maturation and differentiation into plasma cells [19]. Structurally, BCMA is comprised of three major domains: extracellular (amino acids 1–54, linked by disulfide bonds at positions 8–21, 24–37, and 28–41), transmembrane (amino acids 55–77), and cytoplasmic (amino acids 78–184). In normal human tissues, both BCMA protein and mRNA are found almost exclusively on plasma cells and are selectively overexpressed during plasmacyte malignant transformation, promoting tumor cell growth, survival, and drug resistance, primarily through activation of the NFκB, AKT, phosphoinositide 3-kinase (PI3K), STAT3, and MAPK intracellular signal transduction cascades [20,21,22,23,24]. This consistent elevation and virtually sole confinement of BCMA on the surface of MM cells from both cell lines and patient samples has made BCMA a compelling target for drug discovery and development in MM [25,26]. In addition, several studies have provided substantial evidence pointing to the value of using membrane bound BCMA measurements not only as a biomarker for MM diagnosis and prognosis, but also as a possible predictor of response to treatment [27]. Moreover, the finding that BCMA is expressed at similar levels during the various stages of MM, from previously untreated to relapse, suggests that BCMA may be a valid therapeutic target throughout the course of the disease [28].

Blood levels of a soluble form of BCMA (sBCMA), the result of the shedding of BCMA from the plasma cell surface due to cleavage by γ-secretase [29], have been shown to be elevated in MM patients and are linked to inferior clinical outcomes [30]. sBCMA, which is comprised of the extracellular domain plus a portion of the transmembrane domain of BCMA [29], not only lowers the density of the target antigen but also provides a soluble decoy capable of limiting the efficacy of anti-BCMA agents currently under development. This potential hurdle has stimulated the search for γ-secretase inhibitors, which already have attained a prominent role in the quest for drugs to treat Alzheimer’s disease and a number of Notch-overexpressing cancers [31,32], as well as to enhance outcomes in BCMA-directed therapies [33,34].

## 4. Anti-BCMA Monoclonal Antibodies

A core feature of IgG antibodies is the presence of an abundance of fucosyl groups in the N-linked biantennary complex oligosaccharides found in the Fc region [35]. Removal of these groups has become a well-established strategy for enhancing antibody-dependent cellular cytotoxicity (ADCC) through binding to FcγIIIa receptors on natural killer (NK) cells [36,37]. Two such afucosylated anti-BCMA antibodies are described below: the antibody-drug conjugate (ADC) belantamab mafodotin and the naked antibody SEA-BCMA.

Belantamab mafodotin (GSK2857916) is an ADC, in which the antibody is coupled to the microtubule inhibitor monomethylauristatin F (MMAF) through a protease-resistant maleimidocaproyl linker [38]. Binding to the BCMA receptor disrupts BAFF and APRIL signaling to induce ADCC, while the conjugated component produces myeloma cell arrest at the G2/M checkpoint [39]. This immunoconjugate continues to be studied in relapsed and/or refractory MM (RRMM) patients in the DREAMM series of trials (see Table 1). The phase II DREAMM-2 study (NCT03525678) showed that this agent, which has been granted breakthrough therapy and priority review status for RRMM by the FDA, as well as PRIME designation from the European Medicines Agency, has an acceptable safety profile with corneal problems, thrombocytopenia, and anemia, attributed to the MMAF payload, cited as the most commonly observed adverse events. However, the objective response rate (ORR) was only 31% in the DREAMM-2 trial [40] compared to the results of an earlier exploratory study (NCT02064387, DREAMM-1) in which an ORR of 60% was found [41]. Recruitment is currently ongoing for a phase II trial (DREAMM-5; NCT04126200) [42] that includes, along with belantamab mafodotin, two T-cell co-stimulatory agonistic mAbs, GSK3174998 and GSK3359609, which target OX40 [43] and inducible co-stimulator (ICOS) [44,45], respectively. Additionally included in the DREAM-5 trial is a study of the γ-secretase inhibitor nirogacestat (PF-03084014), which blocks the shedding of BCMA from the plasma membrane surface, an approach that has been shown, as stated earlier, to improve effectiveness of anti-BCMA therapy [29]. The conjugate also is under investigation in combination with lenalidomide and bortezomib in a phase II study (NCT03544281; DREAMM-6) [46]. Finally, two phase III studies of belantamab mafodotin recently have been launched: one in combination with pomalidomide (NCT04162210; DREAMM-3) and the other with daratumumab plus bortezomib (NCT04246047; DREAMM-7).

Another afucosylated IgG1 mAb is the humanized antibody from Seattle Genetics known as SEA-BCMA, which has shown promising anti-myeloma activity in pre-clinical models [49] and is the subject of an ongoing phase I study in RRMM (NCT03582033) for which patients currently are being recruited. However, a myeloma-based clinical trial (NCT03266692) of SEA-BCMA in combination with the antibody-coupled T-cell receptor ACTR087 was terminated by the sponsor, following reports of serious adverse effects in an FDA-halted trial that employed ACTR087 and rituximab in a B-cell lymphoma study (NCT02776813) [50].

MEDI2228 is a fully human antibody conjugated to a dimeric minor-groove binding pyrrolobenzodiazepine payload (tesirine) via a protease-cleavable dipeptide (valine-alanine) linker [51]. The conjugate is rapidly internalized and trafficked to the lysosome where the warhead is released leading to DNA damage and subsequent apoptosis. Preclinical studies in mice demonstrated the strong anti-myeloma effects of MEDI2228 even in the presence of clinically significant levels of sBCMA [47,48]. A phase I clinical trial (NCT03489525) has been initiated to determine appropriate dosing of MEDI2228 in RRMM patients, although no data have been reported thus far. CC-99712 is another ADC (composition not available) that recently entered a clinical trial (NCT04036461) for RRMM but results have yet to be described.

## 5. T-Cell-Engaging Bispecific Antibodies

In recent years, T-cell-based antibody therapeutics have assumed an important role in the fight against a number of cancers, including MM. Two main areas of research have dominated this arena: T-cell-engaging bispecific antibodies (T-BsAbs) and chimeric antigen receptor (CAR) T-cell therapies. Based on a concept originally advanced by Nisonoff in the early 1960s [52], T-BsAbs are predicated on the design of a dual-targeting antibody constructed so as to enable one arm initially to bind to the CD3 co-receptor complex on T -cells, while the other arm subsequently is directed to tumor cells via a tumor-associated antigen. The numerous variations on this basic strategy that facilitates recruitment of cytotoxic T-cells to tumor cells in order to effect lysis of the latter recently have been the subject of several extensive reviews [53,54,55,56,57,58]. The cytotoxicity induced by the immunological synapse thus created is due to T-cell release of two cytolytic-initiating proteins—perforin, which produces transmembrane pores in the tumor cell and granzyme B, which navigates through the created pores to initiate apoptosis in the tumor cells [59,60,61,62]. The strategy differs substantially from regular T-cell mediated cytotoxicity in a number of respects. For example, the need for antibody-presenting cells is circumvented, the formation of a major histocompatibility complex (MHC)/antigen complex is not required, and co-stimulatory molecules are not involved. Moreover, such constructs enable “off-the-shelf” use by obviating any requirement for ex vivo T-cell manipulation. Additionally, such constructs produce a polyclonal expansion of T memory cells as a result of persistent T-cell activation. The density of the tumor antigen, as well as the relative binding affinities of each arm for their respective targets and biodistribution, are among the key characteristics of T-BsAbs that impact each construct’s therapeutically relevant properties and which may be fine-tuned to optimize activity [63,64,65,66]. Table 2 lists the currently active clinical trials that include T-cell engaging bispecific antibodies for the treatment of RRMM.

Bi-specific T-cell engagers (BiTEs^®^), developed by Micromet in collaboration with Amgen, represent one type of T-BsAB in which the cross-link is provided by tandem single-chain variable fragments (scFvs) [63,78]. This innovative approach to cancer immunotherapy has borne fruit in the form of the CD3-CD19 cross-linking construct blinatumomab (Blincyto^®^) that was granted accelerated FDA approval in 2014 for use in Philadelphia chromosome-negative B-cell precursor acute lymphocytic leukemia (B-cell ALL), an indication that since has been expanded to include B-ALL patients with minimal residual disease (MRD) [79,80]. Blinatumomab, combined with ASCT, has been the subject of one RRMM-based trial (NCT03173430), which recently was terminated due to “slow patient accrual”. However, the bulk of myeloma-related work using BiTEs has been based on recombinant antibodies to two different epitopes designed to cross-link the CD3ζ chains on the surface of tumor-specific T-cells and the targeted myeloma-BCMA.

Two of the most important and frequently encountered adverse effects that accompany T-cell activating immunotherapies, including those based on T-BsAbs and CAR T-cell formats, are the cytokine release syndrome (CRS; cytokine storm) and neurotoxicity (CAR T-cell-related encephalopathy syndrome; CRES), both of which may be life-threatening [81,82,83]. Almost without exception, CRS is seen in varying degrees of severity in a percentage of participants in every trial of the immunotherapeutic agents described in this and the following section. For example, in an analysis of 15 trials of anti-CD19 or anti-BCMA CAR T-cell constructs, of a total of 977 patients, 62.3% (range: 11% to 100%) experienced some degree of CRS with 18.4% (range 0.8% to 46%) in grades 3 or 4 [82]. Comparisons such as this highlight the problem of assessing the risk of developing CRS and its severity with any specific immunotherapeutic regimen, being subject to influences such as the type of malignancy under study, the structure and target of the immunotherapeutic product involved, and the grading scales used [84].

In its most serious form, the syndrome, which also has been implicated as a serious contributor to the lethal effects of COVID-19 infections [85,86], bears resemblance to a severe inflammatory response. As the name implies, the syndrome has been attributed to the expression and release of various cytokines, most notably IL-6, TNF-α, and IFN-γ [87]. Management of CRS includes corticosteroid infusions and the IL-6 receptor antagonist tocilizumab, which has been approved for the treatment of CAR T-cell associated CRS [88,89]. However, there have been no randomized controlled studies comparing the efficacy of corticosteroids and tocilizumab in the management of CRS. The pathophysiology and management of CRES, which generally occurs within the first two weeks of therapy, have been reviewed by Neelapu [88]. As noted for a few of the products described in subsequent discussions, in some cases the CAR T-cells have been designed to contain a safety switch consisting of a transduced receptor, like CD20 or non-functional truncated epidermal growth factor receptor (EGFR), that can be switched “off” through administration of an antagonist—in these cases, rituximab or cetuximab, respectively—to curtail CAR T-cell toxicity through ADCC and complement dependent cytotoxicity (CDC) [90,91]. Another type of safety switch is based on the dimerization of caspase-9 to activate apoptosis upon exposure to a synthetic dimerizing drug, such as remiducid [92,93,94,95].

AMG 420 (BI-836909), which has been granted fast-track status by the FDA, has shown favorable results in a trial (NCT02514239) of 42 RRMM patients with an overall ORR of 31%, including 70% (7/10) in patients receiving the maximum tolerated dose of 400 g/day. The most serious treatment-related adverse events noted in this study were infections and polyneuropathy. CRS, mostly grade 1, was observed in 38% of patients [67]. AMG 701, whose Fc domain has been engineered to produce a longer serum half-life compared to AMG 420, is currently the subject of a phase I trial (NCT03287908) as monotherapy for RRMM. A preclinical study [68] suggests that a follow-up trial of AMG 701 combined with an immunomodulator may be warranted. REGN5458 (NCT03761108) and REGN5459 (NCT04083534) are two BCMAxCD3 bispecific antibodies developed by Regeneron in partnership with Sanofi. Both are in phase I trials in RRMM patients, although to date preliminary data have been reported only for the former study [69].

Another BCMAxCD3 bispecific antibody teclistamab (JNJ-64007957) has been shown to be well-tolerated in a monkey model [70] and has been included in two clinical trials in RRMM—a phase I dose-escalation study (NCT03145181) and a phase I trial in combination with subcutaneous daratumumab (NCT04108195) plus the CD3xGPRC5D bispecific construct talquetamab. PF-06863135 (PF-3135), a BCMA-CD3 formatted BiTE derived from hinge-mutation engineering of an IgG2a backbone, is presently in a phase I trial (NCT03269136) for RRMM [71,72,73]. Another humanized IgG T-cell engager under clinical scrutiny is CC-93269 (NCT03486067), whose two arms bind in a 2 + 1 format—bivalently to BCMA and monovalently to CD3ε [74].

TNB-383B, under development by Tenebio in collaboration with Abbvie, differs from the other T-BsAbs currently being tested for anti-myeloma activity in that its structure consists of a single immunoglobulin light chain domain in addition to two variable heavy chains. The resulting BCMAxCD3 bispecific format possesses strong T-cell activation kinetics and a low-affinity anti-CD3 arm, resulting in reduced levels of cytokine release while retaining high cytotoxic activity, as demonstrated by studies conducted in vitro and in a mouse xenograft model [75]. TNB-383B, which was granted orphan drug status by the FDA in November 2019, currently is the subject of a phase I trial (NCT03933735) in RRMM [76]. There are a number of other bispecific antibodies that have shown promise for RRMM in preclinical studies. These include TNB-381M [96], FPA-151 [96], EM801 [28], and AP163 [97].

HPN217 provides an example of a tri-specific antibody format. Developed by Harpoon Therapeutics and the subject of a phase I/II trial for RRMM (NCT04184050), HPN217 is comprised of three binding domains in a single chain—an N-terminal BCMA-binding component, a C-terminal single-chain CD3ε TCR-binding portion; and a central domain that binds to human serum albumin. This product’s extended half-life, compared to bispecific formats, has been attributed to its smaller size and flexibility [77].

In addition to BCMAxCD3-based bispecific antibody formats, efforts have been made to develop BCMA-targeted constructs that bind to receptors on NK cells, which, like cytotoxic T-cells, mediate cytotoxicity through release of granzyme and perforin, as well as expression of various apoptosis-inducing ligands [98]. One such construct is a tri-specific product that binds CD16A on NK cells to both BCMA and CD200 on myeloma cells [26,99]. Another is Compass Therapeutics’ CTX-4419, which binds myeloma cell BCMA to both CD16A and p30 on NK cells and has shown some initial promise in preclinical work. Interestingly, NK CD16A engagement is not required for the tumor cell-killing properties of this product [100]. Similar properties have been reported for the related NK cell-engaging multispecific antibodies CTX-8573 [101] and AFM26 [102].

## 6. Chimeric Antigen Receptor (CAR) T-Cells

### 6.1. Autologous CAR T-Cell Therapy

Chimeric antigen receptor (CAR) T-cell therapy has emerged in recent years to a position of prominence in the immunotherapy of cancer [103,104]. This form of adoptive cell transfer (ACT) is designed to convert patient-derived cytotoxic T-cells into specific killers of cancer cells through the use of recombinant DNA techniques by which a viral vector is constructed to express a chimeric receptor against an antigen found on cancer cells. The engineered T-cells are then reinfused into the patient with the intent of lethally attaching to the targeted malignant cells. The technique has been used with success in certain hematological cancers, particularly B-cell malignancies, although solid tumors remain a substantial challenge [105].

The first CAR T-cell products, approved by the FDA in 2017, were tisagenlecleucel (CTL019; Kymriah^®^) and axicabtagene ciloleucel (axi-cel; Yescart^®^), scFv constructs directed against the CD19 antigen, which is uniformly expressed on the surface of malignant B lymphocytes. The former was approved for B-cell acute lymphoblastic leukemia and the latter for diffuse large B-cell lymphoma. The two products differ in a number of respects, such as in the co-stimulatory domain engineered into its chimeric receptor: 4-1BB (CD137; to enhance memory persistence) in tisagenlecleucel and CD28 (to afford greater peak expansion) in axi-cel. In addition, tisagenlecleucel uses a lentiviral vector in the manufacturing process while axi-cel is retroviral-based [106,107,108,109,110,111].

Although ORRs in the range of 80% or higher have been observed in trials of CAR T-cells targeting CD19, durable remissions have been much more difficult to realize [112,113,114,115]. However, numerous studies have demonstrated the benefits of pre-ACT lymphodepletion, usually consisting of fludarabine and cyclophosphamide, in several CAR T-cell-based trials in terms of improved T-cell peak expansion and persistence and clinical outcomes [116]. While lymphodepletion, first shown to be efficacious in malignant melanoma patients [117,118], provides transient tumor control while creating space for CAR T-cell expansion in the bone marrow, the mechanism underlying the improved efficacy putatively attributed to lymphodepletion is not known. Among the proposals advanced to explain the effect are enhanced levels of monocyte chemoattract protein-1 (MCP-1) [119]; elimination of sinks for homeostatic cytokines, such as interleukin-2 (IL-2), IL-7, and IL-15 [120]; and downregulation of indoleamine 2,3-dioxygenase in tumor cells [121].

Whereas CD19 has proven to be a very fruitful target for B-cell malignancies, this has not been the case when applied to MM. As a rule, CD19 is not typically expressed on malignant plasma cells, although it is present on their normal counterparts [122,123]. On the other hand, CD19 has been found to be highly expressed by plasma cells in the bone marrow of MGUS patients, leading to speculation that such cells might be myeloma stem cells [124,125]. However, a trial (NCT02135406) of tisagenlecleucel combined with ASCT produced only a poor clinical benefit in ten MM subjects [126]. Meanwhile, emphasis has shifted to BCMA as a major focus of myeloma-based studies applying the principles of CAR T-cell technology [127,128]. Several of these products, based on the BCMA target, are described below. Table 3 lists the currently active clinical trials that include anti-BCMA directed CAR T-cell constructs.

**Figure 2:**
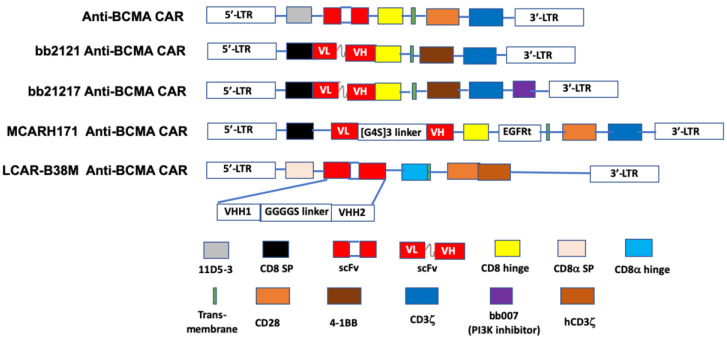
The schematic diagram of representative structures of BCMA-targeted chimeric antigen receptors (CAR). The BCMA CARs contain a single- chain of BCMA antibody variable fragment (ScFv), a transmembrane domain, a hinge region, a co-stimulation domain (4-1BB, CD28 or OX40), and a CD3ζ domain. Additional sequences (such as PI3K inhibitor) are added to enhance identification of CAR+ T cells. LCAR-B38M CAR contains two epitopes of BCMA scFv, VHH1 and VHH2. PI3K: phosphoinositol 3-kinase. Adapted from Lin et al. [156] with permission through Copyright Clearance Center, Inc.

The first in-human study (NCT02215967) of an anti-BCMA CAR T-cell preparation in RRMM was conducted using a lentivirus engineered construct comprised of an anti-BCMA scFv, linked in tandem to a CD8 hinge, a transmembrane region, a co-stimulatory domain (CD28), and CD3ζ as the T-cell activator [156] (Figure 2). An ORR of 81% and a median progression-free survival (PFS) of 31 weeks was reported for 16 patients who received a dose of 9 × 10^6^ T-cells per kg, the highest dose used in the trial. The subjects in this study previously had undergone a median of 9.5 lines of therapy for MM [129].

Idecabtagene vicleucel (bb2121; Ide-cel) is an anti-BCMA scFv fused to the CD137 (4-1BB) co-stimulatory and CD3ζ signaling domains [157]. Following its designation by the FDA as a breakthrough therapy in 2017, bb2121 has been included in a series of phased trials designated KarMMa-1 through KarMMa-4 (NCT03361748 [131], NCT03601078, NCT03651128, and NCT04196491, respectively). Data on the first 33 RR myeloma patients (NCT02658929) [130] in the KarMMa-1 study revealed an ORR of 85%, including 15 patients with complete responses, although six of these subsequently relapsed. The median PFS in this study was 11.8 months. Moreover, 100% (16/16) of evaluable responders in this study with a partial response or better exhibited MRD negativity. Both grade 3 or 4 hematologic (primarily neutropenia) and neurotoxic (42%, all but one of grade 1 or 2) adverse effects were reported. CRS was noted in 76% of the patients. A press release from BMS and bluebird bio, the drug’s sponsors [132], reported data on the KarMMa-2 trial that included 128 evaluable RRMM patients, who were administered either 150, 300, or 450 × 10^6^ CAR T-cells. A median PFS of 8.6 months was found and the toxicity profile was similar to that reported in the earlier phase. Based on these data, a Biologic License Application was submitted to the FDA in early 2020 [158].

A next-generation construct is bb21217, which employs the same lentiviral design as bb2121 but to which an extra PI3K inhibitor domain (bb007) has been added during ex vivo culturing. This production modification has been shown to significantly enhance CAR T-cell-based immunotherapy by enriching the final product’s population of memory-like T-cells, thereby augmenting T-cell durability and potency [159]. Currently, bb21217 is the subject of a phase I dose-escalation trial (NCT03274219) in RRMM patients. Adverse event data have been reported for 22 patients thus far in this trial—13 developed CRS while 5 experienced neurotoxicity and both toxicities were reported resolved. Clinical responses were observed in 15 of 18 evaluable patients, although 6 subsequently experienced relapse. Persistence of CAR T-cells was detected in six of the eight patients evaluated at six months, while two patients had detectable levels after 18 months [133].

JNJ-68284528 (LCAR-B38M; JNJ-4528) is unique among BCMA-targeted T-cell therapies in that it is directed against two BCMA epitopes (VH1 and VH2) to confer improved affinity for BCMA-expressing cells. Data on 57 patients, who had received an average of three prior therapies, in the phase I LEGEND-2 trial (NCT03090659) of JNJ-68284528 showed an ORR of 88% and PFS of 15 months. CRS, mostly grades 1 or 2, was seen in 83% of subjects [134,135,136]. Reported results from 21 evaluable RRMM patients in the CARTITUDE-1 phase Ib/II trial (NCT03548207) [137] were similar (91% ORR, no PFS given, CRS 88%) to those seen in the LEGEND-2 study. In 2019, as a result of these two trials the EMA accorded JNJ-68284528 PRIME status and the FDA provided this agent with the Breakthrough Therapy Designation. CARTITUDE-2 (NCT04133636) and CARTITUDE-4 (NCT04181827) are additional trials that have been initiated to further study the efficacy of JNJ-68284528 in RRMM patients. Significantly, the latter is a phase III investigation aimed at comparing this CAR-T product with standard triplet therapy.

P-BCMA-101, which received FDA Orphan Drug status in 2019, is a fully humanized anti-BCMA CAR T-cell product, in which a CD3ζ/4-1BB signaling domain is fused to a non-immunoglobulin Centyrin^®^ scaffold. In comparison, such constructs are smaller than those patterned on immunoglobulins, have higher binding affinities, improved stability, reduced immunogenicity, and lower production cost. These qualities are attributed to use of transposon (piggy-BAC^®^)-based technology, instead of a viral vector, in the manufacturing process [160]. Moreover, pre-clinical work showed that the process yields a preponderance of T stem cell memory cells (T_scm_), which offers a potential for therapeutic longevity [161]. A phase I trial (NCT03288493; PRIME) of P-BCMA-101, in 12 heavily pretreated RRMM patients, showed an ORR of 83% in 6 who were evaluated, 1 of whom experienced grade 2 CRS, although additional data more fully supporting the efficacy and safety of this agent have yet to appear [138]. However, according to the drug’s sponsor (Poseida Therapeutics), a 15 year follow-up study has been designed and implemented to explore these issues in depth (NCT03741127) [139]. These latter two trials of P-BCMA-101 also are noteworthy for their inclusion of remiducid as a safety switch apoptosis activator, acting through induction of caspase-9 [92,93,94,95].

JCARH125 and FCARH143 are two fully human svFv bicistronic constructs incorporating the 4-1BB co-stimulatory domain [162]. Both CARs use a lentiviral vector but differ in the method of production. Related to these is MCARH171, a gamma-retroviral engineered product that features a truncated EGFR safety system that can be activated by cetuximab when warranted to mitigate CAR T-cell toxicity [163]. Active clinical trials of these three CAR T-cell products are included in Table 3.

CT053 and CT103A are lentivirus vector-based BCMA-targeted CAR T-cell constructs containing a fully human scFv, a CD8α hinge, as well as transmembrane, 4-1BB co-stimulatory, and CD3ζ activation domains, currently under development by CARSgen Therapeutics. The FDA has granted CT053 the Regenerative Medicine Advanced Therapy (RMAT) designation, based on the initial results of ongoing trials in China (NCT03975907) and the US (NCT03915184) in RRMM patients who previously had been treated with a median of four prior regimens [143,164]. CT103A is being studied in China for RRMM (Chinese Clinical Trial Register ChiCTR1800018137). An ORR of 100% was reported for the first 16 patients who received CT103A in a phase I trial [165].

Among other BCMA-targeted CAR T-cell platforms that recently have entered trials for RRMM are Arcelix’s CART-ddBCMA in which the scFv binding domain is replaced by a proprietary soluble protein antigen-receptor X-linker (sparX) (NCT04155749; 15 year study); Descartes-08, a CD8^+^ T-cell preparation whose co-stimulatory domain remains undisclosed (NCT03448978) [144]; and another CD3-4-1BB product referred to only as CART-BCMA (NCT02546167) [145,146,147].

Both CD19- and BCMA-targeted CAR T-cells have been used in combination in a few trials [150]; for example, NCT03455972 (newly diagnosed MM) [148] and NCT03196414 [149] (RRMM). Three additional trials that use variations on this combination protocol are NCT03549442, NCT03706547, and NCT03767725, although no results have yet been reported for these studies.

A significant departure from the standard scFv design pattern is seen in a BCMA-targeting CAR, designated as FHVH-BCMA-T (FHVH33-CD8BBZ), which contains a fully human heavy-chain-only binding domain (FHVH33). The construct, by eliminating linker-connected light chains, was predicted to reduce the risk of recipient immune responses [166]. The initial results of a trial (NCT03602612) of the drug in 12 patients who had previously received a median of 6 lines of anti-myeloma therapy, showed objective responses in 10 subjects. Although 11 of the 12 patients developed CRS, only 1 was considered grade 3 and that was resolved by tocilizumab [151].

### 6.2. Allogeneic CAR T-Cell Therapy

The generation of short-lived responses, coupled with high risk of CRS and other dose-limiting adverse effects, remains one of the drawbacks of autologously administered BCMA-targeted CAR T-cells in MM. In efforts to mitigate these issues, some CAR T-cell originators have turned their attention toward the potential development of “off-the-shelf” allogeneic products that use T-cells derived from healthy donors. One such example is ALLO-715 whose production process uses the proprietary Transcription Activator-like Effector Nuclease (TALEN^TM^) in a site-specific BCMA gene-editing technique designed to limit T-cell receptor-mediated immune responses in the final product [152,153,154,167]. Following promising pre-clinical results in a murine model, ALLO-715 was advanced to a phase I trial (NCT04093596) for which patient recruitment has been initiated. ALLO-715 is noteworthy for its incorporation of a CD20-based mimotope as a rituximab-enabled safety switch, activation of which may be employed to specifically and efficiently eliminate CAR T-cells through ADCC and complement dependent cytotoxicity (CDC) [91,168] where required to relieve toxicity. This is the first anti-BCMA CAR T-cell product utilizing a CD20-based approach for this purpose. This trial also is noteworthy for its use of an anti-CD52 antibody, ALLO-647, as a selective lymphodepletion agent.

Another allogeneic BCMA-targeted CAR T-cell candidate under development for RRMM is PBCAR269A, a product of Precision BioSciences’ proprietary ARCUS^®^ nuclease gene-editing platform, which is predicated on the homing endonuclease I-CreI scaffold [155,169]. In 2020, PBCAR269A was advanced to a myeloma-based phase I trial (NCT04171843) based on data presented recently on two other products in the company’s allogeneic CAR T-cell portfolio on other cancers: PBCAR0191 (NCT03666000) and PBCAR20A (NCT04030195), which target CD19 and CD20, respectively.

Finally, encouraging preclinical data recently have appeared that portends the possibility of engineering CARs that employ T-cell alternatives, such as NK cells [170,171], to generate novel systems targeting BCMA for future allogeneic product development in the quest for innovative anti-myeloma treatments.

## 7. Summary and Future Prospects

The armamentarium of available treatment modalities for MM, formerly restricted to alkylating agents and corticosteroids, has changed dramatically in the past two decades with the introduction of agents working by novel mechanisms, such as proteasome inhibition, immunomodulation, and CD38 blockade. The collective effect has been a remarkable improvement in the disease’s five-year survival rate. However, this success is tempered by the almost invariable relapse and refractoriness to treatment that eventually emerge. This has spurred an ongoing quest for innovative targets subject to control through new drug discovery efforts, especially in the relapsed/refractory setting. One such target is BCMA, whose virtually exclusive confinement to malignant plasma cells has attracted much attention as an immunotherapeutic point of attack in myeloma-based new drug development. In this there is some reason for optimism.

In support of that positive outlook is the BCMA-targeting ADC belantamab mafodotin (GSK2857916), the subject of two recently launched phase III trials wherein the drug is combined with either pomalidomide or daratumumab plus bortezomib. The results of these and other future trials will help determine the eventual role of ADCs in myeloma-based therapeutics. In addition to its comparative simplicity of manufacture and consequential cost-savings benefit, a significant advantage of this ADC over other BCMA-targeted immunotherapies is that it carries virtually no risk of potentially lethal CRS.

Although several T-cell engaging bispecific antibodies targeting BCMA have been developed for treating MM, to date none has been included in myeloma studies beyond early phase trials. The most advanced member of this group, the BiTE construct AMG 420, has shown some encouraging efficacy but suffers from the requirement for continuous intravenous infusion although intermittent dosing is being investigated [67]. The related BiTE product, AMG 701, with its extended half-life and potential for once-weekly dosing may offer a more suitable alternative for future large-scale development in this drug class [68]. Several additional T-cell engaging products with similarly favorable pharmacokinetics recently have entered RRMM trials.

Most of the current interest in the prospects for immunotherapy in RRMM lies in CAR T-cell technology. Since the successful 2017 introduction of two CD19-targeting CAR T-cell products for certain B-cell leukemias and lymphomas (tisagenlecleucel and axicabtagene ciloleucel), there has been a flurry of activity geared toward the development of similar type constructs targeting BCMA. At this point, the two leading candidates appear to be Idecabtagene vicleucel (bb2121) and JNJ-68284528 (LCAR-B38M), both of which are in phase III trials. In the meantime, a number of products in this class continue to enter the pipeline each year and the flow shows no sign of abating. As the field continues to mature and engineering and manufacturing technologies evolve toward optimization and planning for future trials of these products moves forward, serious challenges need to be addressed if CAR T-cell technology is to take its place in the spectrum of accepted regimens available to treat MM, not only in the relapsed/refractory stage but perhaps eventually in earlier lines of therapy for the disease. Investigations aimed at informing dose levels compatible with maximizing response depth and durability while reducing adverse effects, especially CRS, continue apace. In addition, studies of additional efficacy determinants, such as kill-switch design and employment and the role, mode, and mechanism of lymphodepletion, will continue to determine the utility of CAR T-cell therapeutics in RRMM going forward. In the background looms the question of how the process can be made applicable to allogeneic products that offer off-the-shelf advantages, even as a few attempts to develop such products have begun to appear on the scene. The challenges ahead are formidable but the current level of activity in the myeloma-based CAR T-cell therapeutics arena augurs well for a promising future in which BCMA will continue to play a major role as a highly attractive target for conceptualizing new drug design and development, as well as a potential cure for this unrelenting disease.

## Figures and Tables

**Table 1:** Active trials of anti- B cell maturation antigen (BCMA) monoclonal antibodies and their drug conjugates in relapsed/refractory multiple myeloma (RRMM).

Trial ID [References]	Treatment	Phase	Enrollment	Trial Title
NCT02064387 [41]	Belantamab mafodotin (GSK2857916)	I	79	A Phase I Open-label, Dose Escalation Study to Investigate the Safety, Pharmacokinetics, Pharmacodynamics, Immunogenicity and Clinical Activity of the Antibody Drug Conjugate GSK2857916 in Subjects With Relapsed/Refractory Multiple Myeloma and Other Advanced Hematologic Malignancies Expressing BCMA (DREAMM 1)
NCT03525678 [40]	Belantamab mafodotin (GSK2857916)	II	221	A Phase II, Open Label, Randomized, Two-Arm Study to Investigate the Efficacy and Safety of Two Doses of the Antibody Drug Conjugate GSK2857916 in Participants With Multiple Myeloma Who Had 3 or More Prior Lines of Treatment, Are Refractory to a Proteasome Inhibitor and an Immunomodulatory Agent and Have Failed an Anti-CD38 Antibody (DREAMM 2)
NCT04162210	Belantamab mafodotin (GSK2857916) + Pom + low dose Dex	III	380	A Phase III, Open-Label, Randomized Study to Evaluate the Efficacy and Safety of Single Agent Belantamab Mafodotin Compared to Pomalidomide Plus Low dose Dexamethasone (Pom/Dex) in Participants with Relapsed/Refractory Multiple Myeloma (RRMM) (DREAMM 3)
NCT03848845	Belantamab mafodotin (GSK2857916) + Pemb	II	40	A Phase I/II Single Arm Open-Label Study to Explore Safety and Clinical Activity of GSK2857916 Administered in Combination With Pembrolizumab in Subjects With Relapsed/Refractory Multiple Myeloma—DREAMM 4
NCT04126200 [42]	Belantamab mafodotin (GSK2857916) + GSK3174998 + GSK3359609 + Nirogacestat	II	464	A Phase I/II, Randomized, Open-label Platform Study Utilizing a Master Protocol to Study Belantamab Mafodotin (GSK2857916) as Monotherapy and in Combination With Anti-Cancer Treatments in Participants with Relapsed/ Refractory Multiple Myeloma (RRMM)—DREAMM 5
NCT03544281 [46]	Belantamab mafodotin (GSK2857916) + Len + Dex + Bort	II	123	A Phase I/II, Open-label, Dose Escalation and Expansion Study to Evaluate Safety, Tolerability, and Clinical Activity of the Antibody-Drug Conjugate GSK2857916 Administered in Combination With Lenalidomide Plus Dexamethasone (Arm A), or Bortezomib Plus Dexamethasone (Arm B) in Participants With Relapsed / Refractory Multiple Myeloma—DREAMM 6
NCT04246047	Belantamab mafodotin (GSK2857916) + Dara + Bort + Dex	III	478	A Multicenter, Open-Label, Randomized Phase III Study to Evaluate the Efficacy and Safety of the Combination of Belantamab Mafodotin, Bortezomib, and Dexamethasone (B-Vd) Compared With the Combination of Daratumumab, Bortezomib and Dexamethasone (D-Vd) in Participants With Relapsed/Refractory Multiple Myeloma—DREAMM 7
NCT03582033	SEA-BCMA + Dex	I	185	A Phase 1 Study of SEA-BCMA in Patients with Relapsed or Refractory Multiple Myeloma
NCT03489525 [47,48]	MEDI2228	I	106	A Phase 1, Open-label Study to Evaluate the Safety, Pharmacokinetics, Immunogenicity, and Preliminary Efficacy of MEDI2228 in Subjects with Relapsed/Refractory Multiple Myeloma
NCT04036461	CC-99712	I	120	A Phase 1, Multicenter, Open-label, Dose Finding Study of CC-99712, a BCMA Antibody-Drug Conjugate, in Subjects with Relapsed and Refractory Multiple Myeloma

Abbreviations: Bort = bortezomib; Dara = daratumumab; Dex = dexamethasone; Len = lenalidomide; Pemb = pembrolizumab; Pom = pomalidomide.

**Table 2:** Active trials of T-cell-engaging bispecific antibodies in RRMM.

Trial ID [References]	Treatment	Phase	Enrollment	Trial Title
NCT02514239 [67]	AMG 420 (BI 836909)	I	43	An Open Label, Phase I, Dose Escalation Study to Characterize the Safety, Tolerability, Pharmacokinetics, and Pharmacodynamics of Intravenous Doses of BI 836909 in Relapsed and/or Refractory Multiple Myeloma Patients
NCT03287908 [68]	AMG 701	I/II	270	A Phase 1/2 Open-label Study Evaluating the Safety, Tolerability, Pharmacokinetics, Pharmacodynamics, and Efficacy of AMG 701 in Subjects with Multiple Myeloma (ParadigMM-1B)
NCT03761108 [69]	REGN5458	I/II	74	Phase 1/2 FIH Study of REGN5458 (Anti-BCMA x Anti-CD3 Bispecific Antibody) in Patients with Relapsed or Refractory Multiple Myeloma
NCT04083534	REGN5459	I/II	56	Phase 1/2 FIH Study of REGN5459 (Anti-BCMA x Anti-CD3 Bispecific Antibody) in Patients with Relapsed or Refractory Multiple Myeloma
NCT03145181 [70]	Teclistamab (JNJ-64007957)	I	160	A Phase 1, First-in-Human, Open-Label, Dose Escalation Study of JNJ-64007957, a Humanized BCMA x CD3 DuoBody^®^ Antibody in Subjects with Relapsed or Refractory Multiple Myeloma
NCT04108195	Daratumumab + Talquetamab + Teclistamab (JNJ-64007957)	I	100	A Phase 1b Study of Subcutaneous Daratumumab Regimens in Combination With Bispecific T Cell Redirection Antibodies for the Treatment of Subjects with Multiple Myeloma
NCT03269136 [71,72,73]	PF-06863135	I	80	A Phase I, Open Label Study to Evaluate the Safety, Pharmacokinetic, Pharmacodynamic and Clinical Activity of Pf-06863135, a B-Cell Maturation Antigen (BCMA)-CD3 Bispecific Antibody, In Patients with Relapsed/Refractory Advanced Multiple Myeloma (MM)
NCT03486067 [74]	CC-93269	I	120	A Phase 1, Open-label, Dose Finding Study of CC-93269, a BCMA X CD3 T Cell Engaging Antibody, in Subjects with Relapsed and Refractory Multiple Myeloma.
NCT03933735 [75,76]	TNB-383B	I	72	A Multicenter, Phase 1, Open-label, Dose-escalation and Expansion Study of TNB-383B, a Bispecific Antibody Targeting BCMA in Subjects with Relapsed or Refractory Multiple Myeloma
NCT04184050 [77]	HPN217	I/II	70	A Phase 1/2 Open-label, Multicenter, Dose Escalation and Dose Expansion Study of the Safety, Tolerability, and Pharmacokinetics of HPN217 in Patients With Relapsed/Refractory Multiple Myeloma

**Table 3:** Active trials of BCMA-targeted chimeric antigen receptor T-Cells in RRMM.

Trial ID [References]	Treatment	Phase	Enrollment	Trial Title
NCT02215967 [129]	Anti-BCMA-CAR T cells + Ctx + Flu	I	30	A Phase I Clinical Trial of T-Cells Targeting B-Cell Maturation Antigen for Previously Treated Multiple Myeloma
NCT03502577 [34]	Anti-BCMA-CAR T cells + Ctx + Flu + LY3039478 (gamma-secretase inhibitor)	I	18	A Phase I Study of B-Cell Maturation Antigen (BCMA)-Specific Chimeric Antigen Receptor T Cells in Combination With JSMD194, a Small Molecule Inhibitor of Gamma Secretase, in Patients With Relapsed or Persistent Multiple Myeloma
NCT02658929 [130]	bb2121	I	67	A Phase 1 Study of bb2121 in BCMA-Expressing Multiple Myeloma (CRB-401)
NCT03361748 [131]	Idecabtagene vicleucel (bb2121)	II	149	A Phase 2, Multicenter Study to Determine the Efficacy and Safety of bb2121 in Subjects with Relapsed and Refractory Multiple Myeloma (KarMMa-1)
NCT03601078 [132]	Idecabtagene vicleucel (bb2121)	II	181	A Phase 2, Multicohort, Open-label, Multicenter Study to Evaluate the Efficacy and Safety of bb2121 in Subjects With Relapsed and Refractory Multiple Myeloma and in Subjects with Clinical High-Risk Multiple Myeloma (KarMMa-2)
NCT03651128	Idecabtagene vicleucel (bb2121) + standard MM regimens	III	381	A Phase 3, Multicenter, Randomized, Open-label Study to Compare the Efficacy and Safety of bb2121 Versus Standard Regimens in Subjects with Relapsed and Refractory Multiple Myeloma (RRMM) (KarMMa-3)
NCT04196491	Idecabtagene vicleucel (bb2121) + Carf + Ctx + Flu + Len	I	60	A Phase 1, Open-label, Multicenter Study to Evaluate the Safety of bb2121 in Subjects with High Risk, Newly Diagnosed Multiple Myeloma (KarMMa-4)
NCT02786511	Idecabtagene vicleucel (bb2121)	--	50	Longterm Follow-up of Subjects Treated With bb2121
NCT03274219 [133]	bb21217	I	74	A Phase 1 Study of bb21217, an Anti-BCMA CAR T Cell Drug Product, in Relapsed and/or Refractory Multiple Myelom
NCT03090659 [134,135,136]	JNJ-68284528 (LCAR-B38M)	I/II	100	A Clinical Study of Legend Biotech BCMA-chimeric Antigen Receptor Technology in Treating Relapsed/Refractory (R/R) Multiple Myeloma Patients (LEGEND-2)
NCT03548207 [137]	JNJ-68284528 (LCAR-B38M)	I/II	118	A Phase 1b-2, Open-Label Study of JNJ-68284528, A Chimeric Antigen Receptor T-Cell (CAR-T) Therapy Directed Against BCMA in Subjects with Relapsed or Refractory Multiple Myeloma (CARTITUDE-1)
NCT04133636	JNJ-68284528 (LCAR-B38M) + Len	II	80	A Phase 2, Multicohort Open-Label Study of JNJ-68284528, a Chimeric Antigen Receptor T Cell (CAR-T) Therapy Directed Against BCMA in Subjects with Multiple Myeloma (CARTITUDE-2)
NCT04181827	JNJ-68284528 (LCAR-B38M) + Pom + Bort + Dex + Dara	III	400	A Phase 3 Randomized Study Comparing JNJ-68284528, a Chimeric Antigen Receptor T Cell (CAR-T) Therapy Directed Against BCMA, Versus Pomalidomide, Bortezomib and Dexamethasone (PVd) or Daratumumab, Pomalidomide and Dexamethasone (DPd) in Subjects with Relapsed and Lenalidomide-Refractory Multiple Myeloma (CARTITUDE-4)
NCT03288493 [138]	P-BCMA-101 + Rimiducid	I/II	220	Open-Label, Multicenter, Phase 1 Study to Assess the Safety of P BCMA-101 in Subjects with Relapsed / Refractory Multiple Myeloma (MM) Followed by a Phase 2 Assessment of Response and Safety (PRIME)
NCT03741127 [139]	P-BCMA-101 + Rimiducid	I	100	Open Label, Multicenter, Long-Term Follow-Up Study for Subjects Treated With P-BCMA-101
NCT03070327 [55,140]	MCARH171 + Ctx + Len	I	20	A Phase I Trial of B-cell Maturation Antigen (BCMA) Targeted EGFRt/BCMA-41BBz Chimeric Antigen Receptor (CAR) Modified T Cells With or Without Lenalidomide for the Treatment of Multiple Myeloma (MM)
NCT03338972 [55,141]	FCARH143 + Ctx + Flu	I	25	A Phase I Study of Adoptive Immunotherapy for Advanced B-Cell Maturation Antigen (BCMA)+ Multiple Myeloma With Autologous CD4+ and CD8+ T Cells Engineered to Express a BCMA-Specific Chimeric Antigen Receptor
NCT03430011 [55,142]	JCARH125	I/II	245	An Open-Label Phase 1/2 Study of JCARH125, BCMA-targeted Chimeric Antigen Receptor (CAR) T Cells, in Subjects With Relapsed or Refractory Multiple Myeloma
NCT03975907 [143]	CT053	I/II	62	An Open Label, Phase I/II Clinical Trial to Evaluate the Safety and Efficacy of Fully Human Anti-BCMA Chimeric Antibody Receptor Autologous T Cell (CAR T Infusion in Patients With Relapsed and/or Refractory Multiple Myeloma
NCT03915184 [143]	CT053	I	70	Open Label, Multi-center, Phase 1b/2 Clinical Trial to Evaluate the Safety and Efficacy of Autologous CAR BCMA T Cells (CT053) in Patients With Relapsed and/or Refractory Multiple Myeloma
NCT04155749	CART-ddBCMA	I	12	Master Protocol for the Phase 1 Study of Cell Therapies for the Treatment of Patients With Relapsed Refractory Multiple Myeloma, Including Long-term Safety Follow-up
NCT03448978 [144]	Descartes-08 + Ctx + Flu	I/II	30	Combined Phase I-Phase II Study of Autologous CD8+ T-cells Transiently Expressing a Chimeric Antigen Receptor Directed to B-Cell Maturation Antigen in Patients With Multiple Myeloma
NCT02546167 [145,146,147]	CART-BCMA	I	25	Pilot Study Of Redirected Autologous T Cells Engineered To Contain an Anti-BCMA scFv Coupled To TCRζ And 4-1BB Signaling Domains in Patients With Relapsed and/or Refractory Multiple Myeloma
NCT03455972 [148]	CART-anti-CD19/BCMA	I/II	15	Study of T Cells Targeting CD19/BCMA (CART-19/BCMA) for High Risk Multiple Myeloma Followed With Auto-HSCT
NCT03196414 [149]	CART-anti-CD19/BCMA	I/II	10	Study of T Cells Targeting CD138/BCMA/CD19/More Antigens (CART-138/BCMA/19/More) for Chemotherapy Refractory and Relapsed Multiple Myeloma
NCT03549442 [150]	BCMA CART + huCART19	I	39	Phase 1 Study of CART-BCMA With or Without huCART19 as Consolidation of Standard First or Second-Line Therapy for High-Risk Multiple Myeloma
NCT03706547 [150]	Anti-CD19/BCMA CAR-T cells	I	20	Clinical Study of Anti-CD19/BCMA Bispecific Chimeric Antigen Receptors (CARs) T Cell Therapy for Relapsed and Refractory Multiple Myeloma
NCT03767725 [150]	Anti-BCMA or/and Anti-CD19 CAR Autologous T Cells	I	10	Phase I Trial Study of Anti-BCMA (B-cell Maturation Antigen) or/and Anti-CD19 Chimeric Antigen Receptor T Cells (CART Cell) Treatment for the Patient of Relapsed Multiple Myeloma
NCT03602612 [151]	Anti-BCMA CAR T cells + Ctx + Flu	I	42	A Phase I Clinical Trial of T Cells Expressing a Novel Fully-human Anti-BCMA CAR for Treating Multiple Myeloma
NCT04093596 [152,153,154]	ALLO-715 + ALLO-647 + Ctx + Flu	I	90	A Single-Arm, Open-Label, Phase 1 Study of the Safety, Efficacy, and Cellular Kinetics/ Pharmacodynamics of ALLO-715 to Evaluate an Anti-BCMA Allogeneic CAR T Cell Therapy in Subjects With Relapsed/Refractory Multiple Myeloma (UNIVERSAL)
NCT04171843 [155]	PBCAR269A + Ctx + Flu	I/II	48	A Phase 1/2a, Open-label, Dose-escalation, Dose-expansion Study to Evaluate the Safety and Clinical Activity of PBCAR269A in Study Participants With Relapsed/Refractory Multiple Myeloma

Abbreviations: Bort = bortezomib; Carf = carfilzomib; Ctx = cyclophosphamide; Dara = daratumumab; Dex = dexamethasone; Flu = fludarabine; Len = lenalidomide; Pom = pomalidomide.
